# Six-year trend analysis of malaria prevalence at University of Gondar Specialized Referral Hospital, Northwest Ethiopia, from 2014 to 2019

**DOI:** 10.1038/s41598-022-05530-2

**Published:** 2022-01-26

**Authors:** Amanuel Mulugeta, Atsede Assefa, Atsede Eshetie, Birhanie Asmare, Meseret Birhanie, Yemataw Gelaw

**Affiliations:** School Biomedical and Laboratory Sciences, College of Medicine and Health Sciences, The University of Gondar, Gondar, Ethiopia

**Keywords:** Immunology, Microbiology, Molecular biology, Biomarkers, Diseases, Medical research, Molecular medicine

## Abstract

Globally, malaria is the major public health disease caused by plasmodium species and transmitted by the bite of the female anopheles mosquito. Assessment of the trend of malaria prevalence is important in the control and prevention of the disease. Therefore, the objective of this study was to assess the six year trend of malaria prevalence at the University of Gondar Comprehensive Specialized Hospital, northwest Ethiopia, from 2014 to 2019. A retrospective laboratory registration logbook review study was conducted on the malaria blood film examination results at the University of Gondar Comprehensive Specialized Hospital. The data was collected by using a data extraction tool and entered into SPSS version 20 for analysis. Descriptive statistics were used to summarize the socio-demographic characteristics of study participants and presented by graphs, tables and texts. The binary logistic regression was also used to test the association the trend of malaria prevalence and different factors like sex, age, year, and season. From a total of 17,500 malaria blood film examinations, 1341 (7.7%) were confirmed for malaria parasites. Of the confirmed malaria cases, 47.2%, 45.6% and 7.2% were *P. vivax, P. falciparum* and mixed infection*,* respectively. The proportion of *P. vivax* was the predominant species in the first three study years (2014–2016) and *P. falciparum* became the predominant species in the last three study years (2017–2019). The odds of malaria prevalence was lower by 68%, 60% and 69% in the year 2017, 2018 and 2019 compared to 2014, respectively. It was also 1.41 times higher in males than in females. Moreover, the odds of malaria prevalence were 1.60, 1.64, 2.45 and 1.82 times higher in the age group of < 5, 5–14, 15–24 and 25–54 years old compared to the older age groups (> 54 years old), respectively. Even there was a significant declining in prevalence trend; malaria is still a major public health problem. The study showed that there was high seasonal fluctuation from year to year. Moreover, males and the younger age groups were more affected than females and old age groups, respectively. Therefore, malaria prevention and control activities should be strengthened and require extra efforts by considering these variability.

## Introduction

Malaria is one of the protozoan blood parasite that cause morbidity and mortality globally^1^. It is a major public health problem throughout human history, particularly in the tropical and subtropical parts of the world.

According to records from the Ethiopian Federal Ministry of Health, 75% of the country is malarious at which 68% of the total population is living^2^. Malaria is very severe and leading cause of morbidity and mortality for many years in Ethiopia^2,3^. There are two peaks seasonal transmissions of malaria in Ethiopia; the months of September to December (autumn) and March to May (spring)^3,4^.

In Ethiopia, including the Amhara region, prevention and control activities of the malaria have been implemented as guided by the National Strategic Plan. These prevention and control activities uses a combination intervention strategy including early diagnosis and prompt treatment, selective vector control that involved use of indoor residual spraying (IRS), insecticide-treated mosquito nets (ITNs) and environmental management^4^.

However, malaria control in the country as a whole and in the region particularly continued to experience many problems. Studies have shown that the Plasmodium species compositions and the number of malaria cases vary over time due to different factors, such as change in weather conditions, intervention measures, environmental or human behavioral risk factors^3,5^. Some studies in Ethiopia revealed that there was a decrement of Plasmodium species over period of years^5,6^. On the other hand, another trend studies showed that there were fluctuation of malaria cases^4,7,8^. So, it is crucial to assess the current trend of malaria prevalence in the country as well as the study area.

Assessment of the pattern of the current malaria prevalence and understanding how malaria varies in the community as a result of seasonal, environmental, geographical or year-to-year changes will help to evaluate the effectiveness of proven control interventions of the disease in a locality^5,9^.

It also gives essential information about achievements of national malaria programs and identifies malaria hot spots. Additionally, it gives important insight into the changing malaria situation, which might guide adjustments of malaria program activities and the prioritization of malaria research and the changing malaria situation requires an updating description of malaria trends^10,11^. Therefore, the objectives of this study were to analyze trends of malaria prevalence at University of Gondar Specialized Referral Hospital, northwest Ethiopia to identify trends of Plasmodium species over the time-period.

## Methods

### Study area and study population

The study was conducted at University of Gondar Comprehensive Specialized Hospital located in Gondar town. Gondar is ancient city which is located Northwest direction of Ethiopia, 727 km away from Addis Ababa, the capital city of Ethiopia and 175 km from Bahir Dar, the capital city of Amhara regional state. The town has latitude and longitude 12°361 N 37°281E with an elevation of 2133 m above sea level. According to Central Statistical Agency of Ethiopia 2015 report, it has twelve sub city and 22 urban and 11 rural kebeles with a projected population of 323,900^12^. The city has 8 public health centers and 1 public comprehensive specialized hospital (University of Gondar Comprehensive Specialized Hospital), more than 13 private clinics and 1 general hospital providing health services like diagnosis, treatment, prevention and control of diseases^13^. All malaria examined blood films at the University of Gondar Comprehensive Specialized Hospital and registered at laboratory registration logbook were source of population. On the other hand, the study population in this study were all malaria examined blood films (including both sexes and any age groups) at the University of Gondar Comprehensive Specialized Hospital for the past 6 years (from 2014 to 2019). All registered malaria blood films, except incomplete data and illegible (unreadable) documents, were included from the study.

### Study design

A retrospective laboratory registration logbook review study was conducted to determine the 6 years trend of malaria prevalence by reviewing malaria blood film examination laboratory registration logbook at laboratory registration log book of University of Gondar Comprehensive Specialized Hospital (2014–2019).

### Sample size and sampling technique

All malaria examined blood films and register at the University of Gondar Comprehensive Specialized Hospital laboratory registration logbook from 2014 to 2019 were the sample size of study. A total of 17,500 malaria examined blood films were included. The malaria examined blood films were selected by the censuses sampling technique.

### Data collection

The six years (2014–2019) malaria blood film examination laboratory registration logbook data was extracted from March to June 2020, at the University of Gondar comprehensive Specialized Hospital laboratory registration log book. The data was collected by laboratory personnel by using data extraction sheet. The data extraction sheet includes result of blood film (Negative and Positive), type of plasmodium species (*P. falciparum*, *P. vivax* and mixed), year of examination, month of examination, season of examination, sex and age of the patient. Data on both negative and positive microscopically confirmed malaria cases were included in the study. At the University of Gondar comprehensive Specialized Hospital, patients presented sign and symptom of malaria (clinical presentation of malaria) were requested by physicians and internists. In Ethiopia, microscopy is the major diagnostic method for malaria, especially in health centers and hospitals^10^. A well-prepared Gimsa stained blood film (both thick and thin smear) was used to diagnose malaria parasites in the laboratory. Unfortunately, complete data regarding clinical presentation of patient, major interventions done against malaria and other environmental factors were not collected.

### Data analysis and interpretation

The data were entered into SPSS version 20 for analysis. Descriptive statistics were used to summarize the socio-demographic of study participants and the frequency of malaria on different independent variables and presented by tables, figures and texts. Multivariable binary logistic regression analyses were performed to determine the association between the dependent (malaria prevalence and independent variables (age, sex, and year and season as categorical variable). The multivariable binary logistic regression model was analyzed with enter method and a *p* value < 0.05 in the multivariable regression model was considered as statistically significant. The model fitness of the final multivariable logistic regression was checked using Hosmer and Lemeshow test.

### Data quality assurance

The data were checked for completeness, cleaned, and sorted daily. Moreover, the data quality was assured by following standard operation procedures, double entry. In addition, the quality of blood film staining reagents (Gimsa) was checked for its expiration date and by running the known blood sample. Moreover, the blood film examination was done by laboratory technologist and Medical parasitologist who had taken training on malaria blood film examination and malaria parasite identification. The laboratory personals are also participated in proficiency test.

### Ethics approval and consent to participate

All methods were performed following the relevant guidelines and regulations. The University of Gondar has an ethical and review committee in each study field to approve the study on humans. Therefore, the ethical clearance of this study was obtained from the Ethical and Review Committee of the School of Biomedical and Laboratory Sciences, College of Medicine and Health Science, University of Gondar. After discussing the purpose and method of the study, verbal consent was obtained from the Medical Director of the University of Gondar Specialized Referral Hospital before the data collection. Since the study was used secondary data from the registration logbook informed consent for the participants was waived by the Ethical and Review Committee of School of Biomedical and Laboratory Sciences, College of Medicine and Health Science, University of Gondar.

## Result

### Characteristics of study participants

During 2014 to 2019, a total of 17,500 malaria blood films (in average 2917 blood films per year) were examined microscopically for malaria diagnosis. More than half of the cases were males, 9542 (55.5%) and this was more or less consistent throughout the six years. In the six trends, the most malaria suspected and examined cases were in the age group of 25–54 (7040 (40.2%)) followed by age group of 15–24 (5540 (31.7%)) and the lowest suspected case was examined in the older age groups (> 54 years old) (1485 (8.5)). The trend of suspected cases (malaria blood film examination) was highly fluctuated. The highest blood film examination was performed in the year of 2015 (2789 (24.1%)) followed by year of 2017 (3348 (19.1%)) (Table 1).

**Table 1:** Socio-demographic characteristics of patients request for malaria examination at University of Gondar Specialized Referral Hospital from 2014 to 2019.

Socio-demographic variable	Year						
Category	2014	2015	2016	2017	2018	2019	Total
**Sex n (%)**							
Female	1072 (47.4)	1900 (45.1)	1258 (45.1)	1540 (46.0)	1314 (42.0)	874 (49.4)	7958 (45.5)
Male	1188 (52.6)	2310 (54.9)	1531 (54.9)	1808 (54.0)	1811 (58.0)	894 (50.6)	9542 (55.5)
**Age in year; n (%)**							
< 5	169 (7.5)	215 (5.1)	246 (8.8)	316 (9.4)	333 (10.7)	300 (17.0)	1579 (9.0)
5–14	227 (10)	400 (9.5)	269 (9.6)	307 (9.2)	317 (10.1)	336 (19)	1856 (10.6)
15–24	753 (33.3)	1545 (36.7)	917 (32.9)	1015 (30.3)	900 (28.8)	410 (23.2)	5540 (31.7)
25–54	931 (41.2)	1719 (40.8)	1106 (39.7)	1412 (42.2)	1274 (40.8)	598 (33.8)	7040 (40.2)
> 54	180 (8)	331 (7.9)	251 (9)	298 (8.9)	301 (9.6)	124 (7)	1485 (8.5)
Total; N (%)	2260 (12.9)	4210 (24.1)	2789 (15.9)	3348 (19.1)	3125 (17.9)	1768 (10.1)	17,500 (100)

### Annual trends of malaria prevalence and proportion of plasmodium species

**Figure 1:**
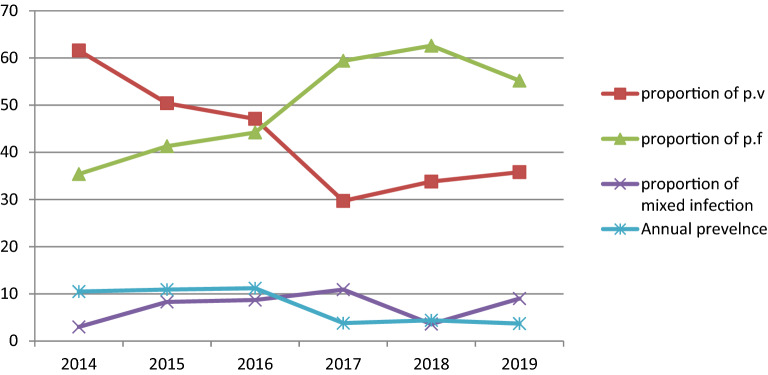
Annual trend of malaria prevalence and proportion of plasmodium species at University of Gondar specialized referral hospital from 2014 to 2019.

Among a total of 17,500 examined blood films, 1341 (7.7%; 95% CI 7.3–8.1) were positive for plasmodium species during the six year period. There were significant fluctuations and reduction trends of overall malaria during the past 6 years, with a maximum of 11.2% and a minimum of 3.7% of cases in 2016 and 2019, respectively. *P. vivax* was the predominant plasmodium species. However, the proportion of the plasmodium species was significantly fluctuated in the six years period (chi squared = 62.58, *p* value < 0.001). In the first 3 study years, the proportion of *P. vivax* was the predominant plasmodium species and in the last 3 study years *P. falcifarum* was the predominant plasmodium species Moreover, mixed infection (*P. vivax* and *P. falcifarum*) showed a significant fluctuating increment trend in the area in the 6 years, with a maximum of 10.9% and a minimum of 3.6% of cases in 2017 and 3 in 2014, respectively (Table 2, Fig. 1).

**Table 2:** Annual trend of malaria prevalence and proportion of plasmodium species in each year among patients requested for malaria examination at University of Gondar Specialized Referral Hospital from 2014 to 2019.

Variable	Total no patient N (%)	Total positivity rate n (%; 95% CI)	Plasmodium species proportion (n = 1341)		
			P.f n (%)	P.v n (%)	Mixed n (%)
**Year**					
2014	2260	237 (10.5; 9.3–11.8)	84 (35.4)	146 (61.6)	7 (3.0)
2015	4210	460 (10.9; 10.0–11.9)	190 (41.3)	232 (50.4)	38 (8.3)
2016	2789	311 (11.2; 10.0–12.4)	137 (44.2)	146 (47.1)	27 (8.7)
2017	3348	128 (3.8; 3.2–4.5)	76 (59.4)	38 (29.7)	14 (10.9)
2018	3125	139 (4.4; 3.8–5.2)	87 (62.6)	47 (33.8)	5 (3.6)
2019	1768	66 (3.7; 2.9–4.7)	37 (55.2)	24 (35.8)	6 (9.0)
Total	17,500	1341 (7.7; 7.3–8.1)	611 (45.6)	633 (47.2)	97 (7.2)

*CI* confidence interval, *P.f* plasmodium falciparum, *P.v* plasmodium vivax.

### Sex, age and seasonal variations of malaria prevalence

**Figure 2:**
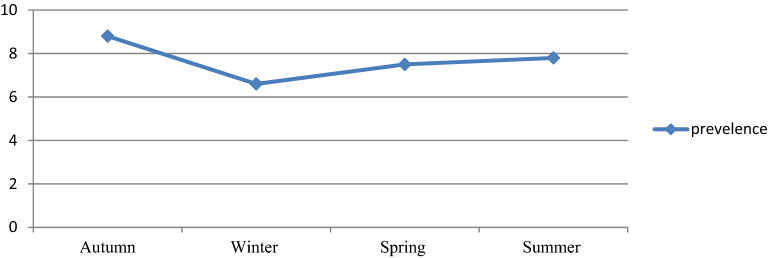
Seasonal variations of malaria prevalence among blood smear microscopy at University of Gondar Specialized Referral Hospital from 2014 to 2019.

**Figure 3:**
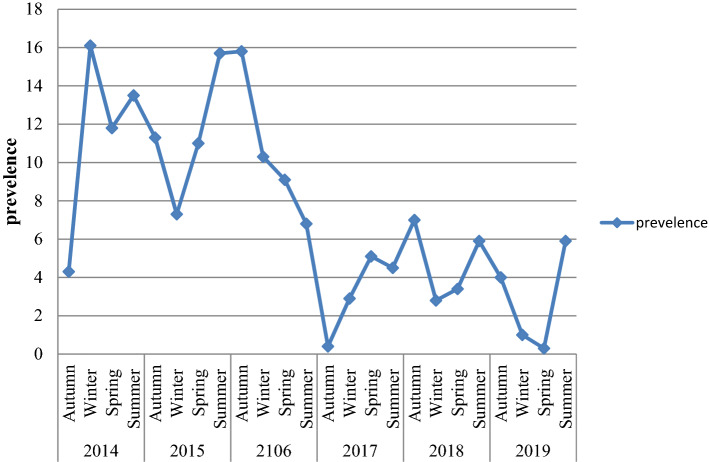
Seasonal variations of malaria prevalence in each year among patients requested for malaria examination at University of Gondar Specialized Referral Hospital from 2014 to 2019.

Despite the apparent fluctuation of total malaria trends over 6 years in the study area, malaria cases occurred throughout the year. However, there was a significant variation between the sexes and different age groups. The odds of malaria prevalence among the male was 1.41(95%CI 1.26–1.59) times higher than females. The prevalence of malaria was also higher in lower age groups compare to the older age groups. The odds of malaria prevalence was 1.60 (95%CI 1.14–2.23), 1.64 (95%CI 1.20–2.26), 2.45 (95%CI 1.86–3.22) and 1.82 (95%CI 1.39–2.40) in the age group of < 5 years, 5–14 years, 15–24 years and 25–54 years, respectively compare to age group of > 54 years old. Controlling of the confounding factors of sex and age, the prevalence of malaria also showed significant reduction in the last 3 study years (2017–2019) compare to the first study year (2014). It was decreased by 68% (95%CI 60–75), 60% (95%CI 51–68) and 69% (95%CI 59–77) in the year of 2017, 2018 and 2019, respectively. Moreover, there was a significant seasonal variation in malaria cases. The highest peak of total malaria positivity rate was observed during autumn, (September, October and November; just after the main rainy season) and the minimum positivity rate was observed during winter (the dry season in the months of December, January and February) and showed significant variation. However, controlling of the sex and age group variation in the season, the highest peak of total malaria positivity rate was observed during summer (June, July and August; main rainy season). Moreover, the seasonal variation was not consistent and highly fluctuated in the six years. Even it was the season where the highest malaria case was reported in over all seasonal malaria prevalence, autumn was the season where lowest malaria case was report in 2014 and 2017 (Table 3, Figs. 2, 3).

**Table 3:** Sex, age and seasonal variations of malaria prevalence and associated factors among patients requested for malaria examination at University of Gondar Specialized Referral Hospital from 2014 to 2019.

Variable	Smear microscopy result		AOR (95% CI)
	Negative n (%)	Positive n (%)	
**Sex**			
Female	7454 (93.7)	504 (6.3)	1
Male	8705 (91.2)	837 (8.8)	1.41 (1.26–1.59)
**Age**			
< 5	1486 (94.1)	93 (5.9)	1.60 (1.14–2.23)
5–14	1736 (93.5)	120 (6.5)	1.64 (1.20–2.26)
15–24	4986 (90)	554 (10.0)	2.45 (1.86–3.22)
25–54	6526 (92.7)	514 (7.3)	1.82 (1.39–2.40)
> 54	1424 (95.9)	61 (4.1)	1
**Year**			
2014	2023 (89.5)	237 (10.5)	1
2015	3750 (89.1)	460 (10.9)	1.02 (0.86–1.20)
2016	2478 (88.8)	311 (11.2)	1.07 (0.89–1.28)
2017	3220 (96.2)	128 (3.8)	0.32 (0.25–0.40)
2018	2986 (95.6)	139 (4.4)	0.40 (0.32–0.49)
2019	1702 (96.3)	66 (3.7)	0.31 (0.23–0.41)
**Season**			
Autumn	3859 (91.2)	374 (8.8)	1
Winter	4002 (93.4)	281 (6.6)	0.84 (0.71–0.99)
Spring	4503 (92.5)	366 (7.5)	1.01 (0.86–1.18)
Summer	3795 (92.2)	320 (7.8)	1.32 (1.12–1.55)

The highest prevalence of malaria was seen in August (9.6%) followed by September and November (9.3%) whereas, the lowest prevalence was seen in January (6.1%). The proportion of Plasmodium species highly fluctuated throughout the 12 months. *Plasmodium vivax* was predominantly high in the winter months (December, January and February), spring months (March, April and May), and the two autumn months (September and November) whereas, Plasmodium falciparum was predominantly high in the summer months (June, July and August) and one of the autumn month (October). The mixed infection was also shoed monthly fluctuation in which the highest peak was observed in March and the lowest peak was observed in December (Table 4).

**Table 4:** Monthly prevalence of malaria and proportion of plasmodium species among patients requested for malaria examination at University of Gondar Specialized Referral Hospital from 2014 to 2019.

Variable	Total no patient N (%)	Total positivity rate n (%)	Plasmodium species proportion (n = 1341)		
			P.f n (%)	P.v n (%)	Mixed n (%)
**Month**					
December	1199	79 (6.6)	36 (45.6)	40 (50.6)	3 (3.8)
January	1551	95 (6.1)	39 (41.1)	49 (51.6)	7 (7.4)
February	1533	107 (7.0)	39 (36.4)	59 (55.1)	9 (8.4)
March	1724	115 (6.7)	37 (32.2)	66 (57.4)	12 (10.4)
April	1633	123 (7.5)	57 (46.3)	58 (47.2)	8 (6.5)
May	1512	128 (8.5)	58 (45.3)	66 (51.6)	4 (3.1)
June	1061	72 (6.8)	47 (65.3)	19 (26.4)	6 (8.3)
July	1422	92 (6.5)	53 (57.6)	33 (35.9)	6 (6.5)
August	1632	156 (9.6)	84 (53.8)	63 (40.4)	9 (5.8)
September	1226	113 (9.3)	43 (38.1)	59 (52.2)	11 (9.7)
October	1719	140 (8.1)	67 (47.9)	63 (45.0)	10 (7.1)
November	1288	120 (9.3)	51 (42.1)	58 (47.9)	12 (9.9)

## Discussion

The present study revealed that the average annual malaria prevalence was 7.7% (95% CI 7.3–8.1). This finding was markedly lower than the study conducted elsewhere in Kola Diba, North Gondar, Northwest Ethiopia (39.6%)^4^, Adi Arkay, North Gondar, Northwest Ethiopia (36.1%)^14^, Abeshge, south-central Ethiopia (33.8%)^5^, Woreta Health Center, Northwest Ethiopia (32.6%)^14^, Dembecha Health Center, West Gojjam Zone, Northwest Ethiopia (16.34%)^8^ and Halaba special district, Southern Ethiopia (9.5%)^15^. However, the current malaria prevalence was higher than other study finding conducted at Felegehiwot Referral Hospital catchment areas, Bahir Dar, northwest-Ethiopia Ethiopia (5%)^7^. The difference might be due to variations in malaria diagnosis quality and the skills of the laboratory personnel to detect and identify malaria parasites. Moreover, the implementation of malaria prevention and control activities might differ from one area to another. Besides, there might be a difference in demographic characteristics (sex, age), geographic location (altitude, temperature, rainfall) and economical activities differences that also had an effect on the prevalence of malaria. The population awareness about malaria bed net application, its transmission, and health seeking behavior might be also different.

The average annual trend of malaria prevalence revealed that there were slight increments in malaria prevalence in the first two years of the study (2015 and 2016) compared to the year 2014, but statistically, it was insignificant. However, in the last three study years (2017, 2018 and 2019) the trend showed a significant reduction in malaria prevalence. The odds of malaria prevalences were reduced by 68%, 60% and 69% in the year 2017, 2018 and 2019, respectively. The possible reasons for malaria reduction during thes study periods (2017–2019) might be due to the increased attention to malaria control and preventive activities by different responsible bodies, increased awareness of the community on the use of ITNs, IRS, the drainage system of mosquito breeding sites and climate change at national and international level. Integrated control strategies are underway in the local area as part of the nationwide malaria control activities^16^. The finding was similar to the 5-year malaria prevalence trend analysis at Dembecha Health Center, West Gojjam Zone, Northwest Ethiopia which reported that there was fluctuated decline of malaria prevalence^8^. However, the observed prevalence in this study was still considerable and public health problem.

This study demonstrated that on average of the six years of study periods, *P. vivax* was the predominant species, although there was a species fluctuation from year to year. The proportion of *P. vivax*, *P. falciparum* and mixed infections was 47.2%, 45.6%, and 7.2%, respectively. This finding was consistent with the study conducted in Adama City, East Shoa Zone, Oromia, Ethiopia^16^, Halaba health center Southern Ethiopia^15^ and Southwest Ethiopia, around Gilgel gibe dam and 10 kilo Metter far from Gilgel gibe dam^3^. The predominance of *P. vivax* might be due to relapse of dormant liver stages or increased treatment pressure against *P. falciparum*^17^. However, this finding was in disagreement with the study conducted at two health centers Gorgora and Chuahit in Dembia district^18^, catchment areas of Felegehiwot Referral Hospital^7^ and Kola Diba, North Gondar, Northwest Ethiopia^4^ which reported that P. *falciparum* was the predominant species. Moreover, the trend of *P. vivax* showed reduction whereas, *P. falciparum* showed an increment trend. In the last three years of the study periods, *P. falciparum* had become the predominant Plasmodium species. The fluctuated proportion of plasmodium species might be attributed to heterogeneous parasite species and disease distribution include differences in genetic polymorphisms underlying parasite drug resistance and host susceptibility, mosquito vector ecology and transmission seasonality. Plasmodium species interact might have geographical differences and these interactions may even change from year to year in a given locale^19^. The finding also revealed that there was fluctuated increment in the proportion of mixed infection.

The prevalence of malaria was varied among different seasons ranging from 6.6 to 8.8%, and these variations were statistically significant. The highest peak was observed in autumn (8.8%) and the lowest peak was observed in the winter season (6.6%). The malaria prevalence was reduced by 16% in the winter. However, where the sex and age were adjusted, the peak prevalence was observed in summer rather than autumn, in which the prevalence was increased by 32%. The reason might be due to climate change from year to year. In Ethiopia, summer is the season when heavy rainfall is observed and it is not a favorable season for vector spreading^16^. However, there is rainfall variation from year to year^20^. Changes in temperature, rainfall, and relative humidity due to climate change are estimated to influence malaria directly by modifying the behavior and geographical distribution of malaria vectors and by changing the length of the life cycle of the parasite. Climate change is also expected to affect malaria indirectly by changing ecological relationships that are important to the organisms involved in malaria transmission (the vector, parasite, and host)^21^.

The current study revealed that males were more affected by malaria infection than females. The odds of malaria positivity rate among males were 1.41 times higher than females. Similar studies showed that males were more affected than females^22–25^. The reason behind the high malaria cases in males might be due to the fact that males are involved in outdoor activities. A study conducted in Dembia district, northwest Ethiopia revealed that individuals involved in outdoor activities were more at risk for malaria infection^25^. The other possible reason might be that males are mobile to malaria-endemic areas seeking temporary employment, whereas females do not perform field activities rather they are cookers and stay at home which might reduce the risk of infection.

Age was also contributing factor to the prevalence of malaria. It was higher in younger age groups than the older age groups. The odds of malaria positivity rate among less than five years old children and 5–14 years old were 1.60 and 1.64 times higher than the age group of greater than 55 years old, respectively. The reason might be these age groups may be less immune to commutate than the older age groups (> 55 years old). This was supported by the world health organization report^26^. The study also showed that the odds of malaria positivity rate among the early working groups (15–24) and primarily working groups (25–54) were, 2.45 and 1.82 times higher than the age group of greater than 55 years old, respectively. Another study, conducted on pregnant women in Sherkole district, Benshangul Gumuz regional state, West Ethiopia also revealed that the older age groups were less likely to have malaria infection^27^. The reason behind the high malaria cases in the mentioned age group of 15–24 and 25–54 years old might be the fact that this age group might be involved in outdoor activities and are mobile to malaria-endemic areas seeking temporary employment, whereas the older age group do not perform field activities rather they are staying at home which might reduce the risk of infection. Moreover, the older age groups might frequently expose to malaria previously, which might develop immunity to malaria infection. It was known that natural infection elicits a robust immune response against the blood stage of the parasite, protecting against malaria^28^. However, according to the studies conducted in rural surroundings of Arba Minch Town, south Ethiopia^29^, and Sudan^30^, age had no significant association with malaria infection. Indeed, these studies were focused on a specific study population; under-five children and pregnant women, respectively.

The finding of the current study had its strengths; one it had enough sample size which increased the power of the study; second, it included all age segments of the populations (from children up to the old age groups). However, this study might suffer from the fact that it is secondary data; the reliability of the recorded data could not be ascertained. Moreover, the collected data relayed on the laboratory logbook which lacks participants’ body temperature, clinical presentations and residence. It also lacks information regarding the weather conditions of the month, seasons and years.

## Conclusion

The current finding showed that there was a significant declining trend of the of malaria prevalence in the study area. However, the overall prevalence was still a major public health problem and requires extra efforts for further reduction. On average, the highest peak of malaria cases was observed during the autumn seasons. However, there was high fluctuation from year to year. Moreover, males, under-five children and the younger age groups were more affected compare to the older age groups. In addition, even *P. vivax* was the predominant Plasmodium species in the allover trend, there was a high fluctuation of Plasmodium species from year to year and season to season. Therefore, prevention and control activities should be continued and strengthened in the study area considering these variabilities.

## Data Availability

All data generated or analyzed during this study are included in this published article.

## Abbreviations

AOR: Adjusted odds ratio
CI: Confidence interval
*P. falciparum*: *Plasmodium Falciparum*
*P. vivax*: *Plasmodium Vivax*
IRS: Indoor residual spraying
ITNs: Insecticide-treated mosquito nets
